# Knowledge of danger signs in newborns and health seeking practices of mothers and care givers in Enugu state, South-East Nigeria

**DOI:** 10.1186/s13052-015-0127-5

**Published:** 2015-03-21

**Authors:** Uchenna Ekwochi, Ikenna K Ndu, Chidiebere DI Osuorah, Ogechukwu F Amadi, Ifeyinwa B Okeke, Ejike Obuoha, Kenechi S Onah, Ikenna Nwokoye, Odutola I Odetunde, Nnenne I Obumneme-Anyim

**Affiliations:** Department of Paediatrics, Enugu State University of Science and Technology, Enugu, Enugu State Nigeria; Child Survival Unit, Medical Research Council UK, The Gambia Unit, Fajara, Gambia; Department of Paediatrics, Nnamdi Azikiwe University Teaching Hospital, Nnewi, Anambra State Nigeria; Department of Paediatrics, Federal Teaching Hospital, Abakaliki, Ebonyi State Nigeria; Department of Paediatrics, University of Nigeria Teaching Hospital, Ituku-Ozara, Enugu State Nigeria

## Abstract

**Background:**

According to UNICEF, 40% of all under-5 deaths occur within the first month of life and half of these within the first few days of life. Many of these deaths are related to late recognition of neonatal illness, delays in decision to seek care at household level and subsequent late intervention at healthcare facilities. Knowledge of mothers about the danger signs in newborn is imperative to reduce these delays and preventable deaths.

**Aim:**

This study aimed to assess the perception of mothers and/or care givers of danger signs in newborns and their knowledge of the WHO recognized danger. A secondary aim was to explore the socio-demographic factors of mothers that influence knowledge of the WHO recognized danger signs and the health seeking behaviors of these mothers and/or care-givers.

**Methods:**

This was a community based descriptive and analytical study which used a multistage sampling technique to select 376 mothers and/or care-givers from four communities in 4 of the 17 Local Government Areas (LGA) of Enugu State. Logistic regression and chi-square was used in testing associations between variables.

**Results:**

Knowledge of more than three of the nine WHO recognized danger sign was poor (0.0-30.3%). Majority of the mothers had knowledge of one (i.e. fever) WHO recognized danger sign (95.2%). Knowledge of the WHO signs was not significantly associated with maternal socio-demographic variables considered in this study. Healthcare seeking behaviour was significantly determined by knowledge of at least one WHO recognized danger sign (OR 4.6 CI 1.1-18.7, *P =* 0.032). Cough, diarrhea and the excessive crying were the most perceived and experienced non-WHO recognized dangers signs among respondents.

**Conclusion:**

There is urgent need to strengthen the teaching and training of expectant mothers across all maternal socio-demographic variables on these danger signs and the most appropriate measures to take when they occur.

## Introduction

The neonatal period (i.e. the first 28 days of life) is the most critical time for the survival of an infant. Despite the fact that global neonatal mortality rate has declined significantly by 40% between 1990 and 2013, this decline has not been in pace with the 56% decline of under-5 mortality rate globally over the same period [1]. When stratified by regions and countries, this slow pace of neonatal mortality decline becomes bleak with the slowest pace of improvement recorded in Sub-Saharan Africa, South and East Asia. For instance, of the 86 million neonatal deaths recorded worldwide between 1990 and 2013, over 65% of them occurred in 10 countries in these regions with India and Nigeria accounting for 25% and 10% of these deaths respectively. According to a report in 2013, almost 2 million newborns died in the first week of life accounting for 73% of all neonatal deaths that year [2]. In Nigeria, the death of newborn babies accounts for more than 28% of all deaths in children before their 5^th^ birthday [3]. Though the highest absolute number of newborn deaths occurs in Asia due to the high birth rate and population in its countries, Africa still accounts for the highest national neonatal mortality rates seen around the world [4].

These disturbing statistics of neonatal mortality occurs because a newborn can die within minutes if prompt recognition, diagnosis and treatment are not initiated [5]. The modified three delays model [6] responsible for newborn death shows that household and health facility related delays were the major contributors to late presentation, treatment initiation and subsequent newborn deaths in many developing countries. These delays especially at the household level are particularly important because once there is a delay in the recognition of the danger signs of newborn illnesses there are automatically delays at all other levels i.e. initiation of appropriate treatment and/or referral to a better resourced hospital etc. Therefore, it becomes necessary to survey the knowledge of the signs which mothers in the developing country may perceive as “danger signs” (signs leading either to recognition of illness or heath care seeking) in the sick newborns. This study sought to determine what mothers in Enugu state perceive as danger signs and their knowledge of the WHO recognized danger signs. It further explored the socio-demographic features of mothers that influence knowledge of these danger signs and the health seeking behaviors of the mothers and/or care-givers.

## Methodology

### Study area

This study was conducted in Enugu state, the host town of Enugu State University of Science and Technology. It is located in south east Nigeria on latitude 6° 27′N and longitude 7° 30′E [7]. The state was nicknamed the coal city because coal mining activities were the driving force behind the city’s growth in the 20th century. The economy of Enugu state is dependent mainly on national oil revenue and commerce. Enugu state is made up of 17 Local Government Areas (LGAs) with its capital carved from Enugu North, Enugu South and Enugu East Local Government Area. The majority of the inhabitants are Igbo by tribe, and Christianity is the dominant religion. The minimum monthly income, similar to the national average is ₦18,000 (110 US$). Literacy rate is 66%, which is higher than the national literacy rate of 45%, and there are 955 males per 1,000 females [8]. The fertility rate and neonatal mortality rate, similar to the national mean, are 4.5 births per woman and 40 per 1,000 live births respectively [8].

### Study subject and sampling technique

This is a community based cross-sectional descriptive and analytical study. The data collection for this study was conducted over a 10 month period (from December 2013 to September 2014) in 4 of the 17 LGAs of Enugu State. Multi-stage sampling method was used to select study participants. The list of the 17 LGAs of Enugu state was obtained from appropriate authorities. In order to obtain a representative sampling, the LGAs were divided into twelve rural and five semi urban categories based on level of development and population in the LGAs [8]. Simple random sampling using balloting method was used to select 2 LGAs from each category.

In the second stage, one community was selected from each LGA using simple random sampling (i.e. one community from each of the two rural and semi-urban LGA). A list of all the communities in the selected LGAs was compiled from the respective local government authorities. The communities of each LGA was numbered and folded into a medium to large sized container. An independent observer not involved in the research was requested to pick one folded paper at a time until four communities were chosen for the selected LGAs. After every pick, the selected paper was discarded and the container was shaken to ensure proper mixing of the remaining folded papers. This process was done separately for each of the 4 selected LGAs.

Based on the crude birth rate of 4.5% and a projected population of 3.5 million people in Enugu state [8] with an anticipated non-response rate of 15% and a 5% margin of error, a minimum of seventy-five (75) mothers were expected to be enrolled for this study. In each of the communities visited, through adequate mobilization using the community leaders, the women were asked to gather at a predetermined. Women who had babies less than 2 years of age and/or had nursed a baby in the past 2 years (in case of care givers) and consented to participate were consecutively enrolled and questionnaires administered. This process was repeated for each of the four communities visited. One hundred and five (105) women and care givers who met the inclusion criteria and gave consent to participate were selected for interview in each of the four communities giving a total of four hundred and twenty respondents from all four communities.

## Measures

### Danger signs in newborns

After obtaining oral informed consent, trained research assistants interviewed the mothers regarding newborn danger signs and actions they took when these danger signs were seen in their newborns. Mothers with children under-2 years old or care-givers who had nursed a child in the last two years where asked to list signs they considered serious health issues and could potentially endanger the life of a newborn. They were also requested to list any of these signs they have personally experienced, the initial actions they took, if they sought help from healthcare institution and reasons for not using healthcare facilities where appropriate. Mothers and/or care-givers were in addition asked to recall the time from noticing the danger sign(s) and presentation to the hospital, promptness of care in the health facility and final outcome of their newborn illness. The danger signs listed by mothers were organized and grouped into World Health Organization recognized danger signs [9] and the respondents perceived danger signs. The “WHO recognized dangers signs” based on WHO definition were categorized as follows: *i)* Not feeding since birth or stopped feeding; *ii)* Convulsion; *iii)* Respiratory rate of 60 or more (fast breathing); *iv)* Severe chest in-drawing (difficulty in breathing); *v)* Temperature of ≥ 37.5 degree centigrade (fever); *vi)* Temperature ≤ 35.5 degree centigrade (hypothermia); *vii)* Only moves when stimulated or not even when stimulated (weakness or lethargy); *viii)* Yellow soles (sign of jaundice); *ix)* Umbilicus redness or draining pus, skin boils, or eyes draining pus (sign of local infection). Based on these, mothers were further categorized into the maximum number of WHO recognized danger signs known and correctly listed (see below).

### Socio-demographic characteristics

*i) Age of respondent:* in years was assessed and grouped as 18–25, 26–30, 31–35 and 36+; *ii) Educational attainment:* was assessed using the highest level of education attained by respondents and grouped into no education, primary, secondary and tertiary education: *iii) Place of residence*: defined as the geographic location of the household was categorized as urban, and rural;; *iv) Marital status:* of the surveyed women was originally grouped as “never married”, “currently married” and “formerly married”. These were re-coded into currently married for mothers in marriage and not married for mothers who have never or formerly married groups. *v) Socioeconomic status:* defined as the wealth index of the household was derived using maternal and paternal highest educational attainment and occupation based on oyedeji classification [10]. This was then categorized as lower, middle and upper class; *vi)* Number of children was defined as the number of children whether dead or alive that mother has given birth to or the number of children a care-giver has nursed prior to this study. They were classified into 1–4 and 4 or more;

### Data cleaning and analysis

Quality control check was done by researchers on daily basis after enrollment and where errors were detected, the interviewers were asked to clarify them accordingly with the interviewed mother or care giver. Microsoft excel 2007 was used to input the raw data. Data cleaning was done by the research assistants and the study researchers. SPSS version 20 was used for data analysis. Chi-square and logistic regression statistical instrument were used to establish the relationship between knowledge of WHO recognized danger signs, mother’s socio-demographic variables and seeking of healthcare service among mothers. Results were presented in percentages, odds ratios and 95% confidence intervals where appropriate. Statistical significance was set at p-value < 0.05.

## Result

### Characteristic of respondents

Four hundred and twenty mothers consented to participate in this study out of which 376 were successfully interviewed giving a recruitment fraction of 90%. Of these, 196 (52.1%) were resident in a rural and 180 (47.9%) resident in a peri-urban area. A majority of the mothers had primary (43.3%) and secondary school (31.8%) as their highest educational attainment while 23 (6.1%) and 72 (19.1%) had no and post-secondary school education respectively. About 4/5^th^ of the mothers (83.8%) were married and most (78.1%) had 4 or fewer children. Slightly over half of the surveyed mothers (51.9%) were in the low socio-economic class with the remaining approximately evenly distributed between the middle (22.5%) and upper (25.7%) socio-economic class (Table 1).

**Table 1:** **Summary description of respondents**

**Variables**	**n (%)**	**Knowledge of danger sign†**		***P-*** **value**
		**None**	**At least 1**	
**Age of Mothers (years)**	**N = 375**			
18-25	80 (21.3)	8 (10.0)	72 (90.0)	0.799
26-30	111 (29.6)	4 (3.6)	107 (96.4)	
31-35	68 (18.1)	2 (3.0)	65 (97.0)	
36+	116 (30.9)	3 (2.6)	112 (97.4)	
**Completed Education**	**N = 376**			
None	23 (6.1)	3 (13.0)	20 (87.0)	0.252
Primary	163 (43.3)	7 (4.3)	154 (96.6)	
Secondary	118 (31.4)	4 (3.4)	114 (96.6)	
Tertiary	72 (19.1)	4 (5.6)	68 (94.4)	
**Place of residence**	**N = 376**			
Rural	196 (52.1)	8 (4.1)	187 (95.9)	0.799
Urban	180 (47.9)	10 (5.6)	169 (94.4)	
**Currently married**	**N = 369**			
No	54 (14.6)	15 (4.8)	299 (95.2)	0.748
Yes	315 (83.8)	2 (3.8)	51 (96.2)	
**Number of children**	**N = 370**			
4 or less	289 (78.1)	15 (5.2)	273 (94.8)	0.307
more than 4	81 (21.9)	2 (2.5)	78 (97.5)	
**Socioeconomic Status**	**N = 374**			
Lower	194 (51.9)	6 (3.1)	187 (96.9)	0.265
Middle	84 (22.5)	4 (4.8)	80 (95.2)	
Upper	96 (25.7)	7 (7.4)	88 (92.6)	
**Experience of ≥ 1 sign in newborn**	**N = 375**			
No	112 (29.9)	10 (3.8)	252 (96.2)	0.292
Yes	263 (70.1)	7 (6.3)	104 (93.7)	
**Knowledge of danger signs** **†**	**N = 376**			
None	18 (4.8)			
At most 1	358 (95.2)			
At most 2	352 (93.6)			
At most 3	296 (78.7)			
At most 4	114 (30.3)			
At most 5	50 (13.2)			
At most 6	11 (29.3)			
At most 7	1 (0.3)			
At most 8	0 (0.0)			
All 9	0 (0.0)			

†WHO recognized danger signs in newborns.

### Danger signs: knowledge, perception and experience

All but one of the mothers surveyed agreed they were aware of some signs in newborn suggestive of imminent danger. When asked to list those signs, 18 (4.8%) had no knowledge of any and listed none. Majority (95.2%) of the mothers listed correctly at least one of the WHO recognized danger signs. Two, three, four and five danger signs were correctly listed by 352 (93.6%), 296 (78.7%), 114 (30.3%) and 50 (13.2%) of the respondents respectively while only 11 (2.9%) and 1 (0.3%) correctly listed up to six and seven WHO recognized danger sign respectively (see Table 1). Two hundred and sixty three (70.1%) of the respondent have or had previously noticed one or more of the danger signs in their current newborns and/or their older children when they were neonates. None of the maternal socio-demographic characteristics was significantly associated with knowledge of at least 1 WHO recognized danger signs (Table 1).

Table 2 shows signs perceived by mothers as danger signs and those that mothers have actually experience in their current or previous newborns. Of the WHO recognized danger signs, fever 305 (25.4%), refusal to feed 102 (8.5%) and weakness 85 (7.1%) were the most frequent signs mentioned as danger signs by the mothers. Others include convulsion 73 (6.1%), “cold body” (i.e. hypothermia) 43 (3.6%), yellowness of the body (i.e. jaundice) 41 (3.4%), difficulty in breathing 35 (2.9%), boil and/or rashes 30 (2.5%) and fast breathing 20 (1.7%). Mothers also mentioned signs perceived as dangers signs which are not WHO recognized. They include but were not limited to diarrhea 123 (10.3%), excessive crying 98 (8.1%), vomiting 81 (6.8%) and abdominal colic 25 (2.1%). Of all mothers who have experienced these signs in their newborns (current or previous), fever 114 (30.9%), convulsion 18 (4.8%) and jaundice 14 (3.7%) were the commonest WHO recognized danger signs that have been experienced by the mothers while cough 29 (7.7%), diarrhea 20 (5.3%) and the excessive crying 14 (3.7%) were the most frequent perceived danger signs experienced by mothers (Table 2).

**Table 2:** **Recognition of the danger signs and consequent healthcare-seeking behavior**

**Signs**	**Danger signs†** ^**1**^ **n (%)**		**Sought medical care subsequent to experience**	
	**Perceived**	**Experienced**	**Yes**	**No**
	**N = 1199**	**N = 263**	**N = 202**	**N = 61**
Weakness †^2^	85 (7.1)	7 (1.9)	5 (71.4)	2 (28.6)
Fever †^2^	305 (25.4)	114 (30.9)	94 (82.5)	20 (17.5)
Excessive crying	97 (8.1)	14 (3.7)	9 (64.3)	5 (35.7)
Vomiting	81 (6.8)	13 (3.5)	11 (86.6)	2 (15.4)
Refusal to feed †^2^	102 (8.5)	4 (1.1)	3 (75.0)	1 (75.0)
Difficulty in breathing †^2^	35 (2.9)	9 (2.4)	8 (88.9)	1 (11.1)
Diarrhea	123 (10.3)	20 (5.3)	14 (70.0)	6 (30.0)
Jaundice †^2^	41 (3.4)	14 (3.7)	11 (78.6)	3 (21.4)
Cough	97 (8.1)	29 (7.7)	23 (79.3)	6 (20.7)
Convulsion †^2^	73 (6.1)	18 (4.8)	13 (72.2)	5 (27.8)
Cold body †^2^	43 (3.6)	2 (0.5)	2 (100.0)	0 (0.0)
Boil and/or rash †^2^	30 (2.5)	8 (2.1)	5 (62.5)	3 (37.5)
Abdominal colic	25 (2.1)	3 (0.8)	1 (33.3)	2 (66.7)
Catarrh	21 (1.8)	4 (1.1)	2 (50.0)	2 (50.0)
Fast breathing †^2^	20 (1.7)	3 (0.8)	1 (33.3)	2 (66.7)
Bulging Fontanel	1 (0.1)	1 (0.3)	0 (0.0)	1 (100.0)
LBW/ Prematurity	2 (0.2)	0 (0.0)	0 (0.0)	0 (0.0)
Inability to stool	5 (0.4)	0 (0.0)	0 (0.0)	0 (0.0)
Abdominal distension	6 (0.5)	0 (0.0)	0 (0.0)	0 (0.0)
Headache (Hotness of head)	7 (0.6)	0 (0.0)	0 (0.0)	0 (0.0)

†^1^minimum and maximum danger signs listed by respondents were 1 and 7 respectively; †^2^ WHO recognized danger signs in newborn [9].

### Danger signs: interventions and health seeking behaviors

Of the 263 mothers and/or care-givers who have experienced the perceived and WHO recognized danger signs in their newborns approximately half of these mothers 125 (47.7%) took the child to the hospital immediately without any home intervention. Five (1.9%) of these mothers did nothing while the remaining 133 (50.5%) did the following: 13 (5.0%) used glucose water, 84 (32.1%) gave over the counter medications (self medication), 12 (4.6%) used local concoction and/or traditional medications and 24 (8.8%) used other interventions. These ‘other’ interventions used included tepid sponging for fever, bathing with rice water for boils/rash, exposure to sunlight for jaundice, rubbing of coconut oil for cold body, giving gripe water for abdominal colic, giving palm-oil for convulsion, applying pepper on body for weak or lethargic newborns to stimulate activity etc. After initial and various intervention 202 (76.8%) and 61 (16.2%) did and did not take their newborn to the hospital respectively. For those who did, close to two-fifth (45%) presented to the hospital more than 24 hours after recognition of the danger signs. More mothers without knowledge of these danger signs presented to hospital later than 24 hours after onset of newborns illness. This however failed to attain statistical significance (*P =* 0.073). Among the 61 mothers who did not present to the hospital at all, the most frequent reasons for not taking newborn to the hospital given by the respondents included; symptoms resolved (40.8%), cost and/or inaccessibility of healthcare facilities (13.1%), lack of knowledge of signs (11.4%) and no reason (29.5%). Others include perceived low care in healthcare facility (1.6%), traditional and/or religious faith (1.6%) and lack of decision autonomy to seek healthcare for newborn (1.6%). Only knowledge of at least one WHO recognized danger sign significantly determined seeking of healthcare in a medical facility in sick newborn (*P =* 0.005) (Table 3). Logistic regression analysis showed that mothers with knowledge of at least one danger sign were more likely to seek healthcare services compared to those with no knowledge of these dangers signs (OR 4.6 CI 1.1-18.7, *P =* 0.032), (Table 3).

**Table 3:** **Analysis of respondent’s healthcare seeking behavior for newborn with danger signs**

**Variables**	**Sought healthcare n (%)**	***P*** **-value**	**Odd ratio OR (95% CI)**	***P-*** **value**
**Mothers age (years)**				
18-25	39 (67.2)	0.181	1.9 (0.7-4.8)	0.196
26-30	62 (81.6)		0.9 (0.4-2.4)	0.892
31-35	34 (73.9)		1.5 (0.6-3.9)	0.429
36+	67(80.7)		1	-
**Completed Education**				
None	9 (60.0)	0.234	1	-
Primary	89 (76.1)		2.8 (0.8-9.7)	0.097
Higher (secondary ± tertiary)	104 (79.4)		1.2 (0.7-2.3)	0.591
**Place of residence**				
Rural	119 (74.8)	0.351	1	-
Urban	83 (79.8)		1.3 (0.5-3.5)	0.652
**Currently married**				
No	34 (82.9)	0.313	1	-
Yes	165 (75.7)		1.8 (0.6-4.9)	0.294
**Number of children**				
4 or less	148 (77.5)	0.676	1	-
more than 4	51 (75.1)		1.5 (0.7-3.3)	0.272
**Socioeconomic Status**				
Lower	116 (75.8)	0.410	1	-
Middle	44 (73.3)		2.8 (0.8-9.7)	0.562
Upper	41 (83.7)		1.2 (0.6-2.3)	0.686
**Knowledge of danger signs**				
None	4 (40.0)	0.005†	1	-
At least 1	197 (78.2)		4.6 (1.1-18.7)	0.032†

† − Statistically significant.

## Discussion

The study revealed that knowledge of at least one danger sign in the newborn considerably increased the likelihood of mother and/or caregiver to seek care in health facilities. Even though the proportion of mothers with at least one danger sign knowledge compared to those with none is skewed towards the former, this is hardly surprising as knowledge of signs signifying imminent danger will in many cases logically evoke a quicker response to seek healthcare services, consequently increasing the chances of child survival. This early response and presentation to the hospital has been documented as an important factor in reduction of neonatal mortality in emergency situation [11,12]. This reiterates the need for renewed and intensive education of mothers about these signs during pregnancy and antenatal care visits in order to minimize the delays in initial household interventions and decision to seek care.

The study further showed that a majority (95.2%) of the respondents had knowledge of one WHO recognized danger sign and those with knowledge of two and three danger signs were also considerably high (93.2% and 78.7% respectively). This high knowledge of few danger signs amongst respondents was corroborated by a similar study in rural India where 67.2% of respondents had knowledge of at least one danger sign in newborns [13]. Though these figures are commendable, the proportion with knowledge of more than three danger signs drastically reduced from 30.3% to 0.3% with none having knowledge of up to 8 or all of the WHO recognized danger signs. A similar reduction in proportion of mothers with knowledge of more than one danger signs in newborns was also noted in studies conducted in Uganda, India and Bangladesh [14-16]. Though there is no universal accepted standard in relation to the number of danger signs that constitute adequate knowledge, it is nonetheless fair to say that knowledge of one or two danger signs out of nine recognized dangers does not suffice for adequate knowledge. The implication of this poor knowledge was reflected in the fraction of mothers with experience of these signs in their sick newborn who sought care in health facilities immediately the danger signs were observed in this study. Less than half (47.7%) presented to the hospital immediately these signs were noticed and about one in four (23%) did not present to the hospital at all following the delays at the household level. This delay will inadvertently result in late presentation to hospital and subsequent late administration of appropriate interventions resulting in mortality. This trend is particularly disturbing especially in Nigeria with one of the worst neonatal mortality rates in the Africa and among the highest in the world.

Perhaps because of the almost non subjective nature and/or the ease of being easily detected, fever, refusal to feed and weakness were and importantly so, the most frequently listed WHO recognized danger signs and also prompted the most healthcare facility visits by respondents. Similarly, cough and diarrhea which are features of the leading cause of child death and potentially fatal illnesses in newborn and excessive crying which could be an unspecific signal for other danger sign in newborns were the most perceived (non-WHO recognized) danger signs that prompted hospital visit among respondents. Other studies [12,14] have documented these signs and others which are non WHO recognized as highly perceived as danger signs amongst mothers. The National Neonatology Forum of India (NNF) recognizing this, has included them as danger signs in its newborn training manual [17]. Though not WHO recognized, some of these signs as identified [18] are indicators of leading causes of morbidity and mortality in newborns and should be considered in any danger sign list in developing countries.

Finally, apart from knowledge of at least one danger sign, most maternal variables recognized as favorable determinants of child survival (i.e. maternal education, age and higher socio-economic status) were not significantly associated with knowledge of WHO designated danger signs and subsequent decision to seek care. There is therefore urgent need to incorporate and/or consolidate the teaching of expectant mothers during ANC on these danger signs. This teaching should specifically focus on how to recognize danger signs and the most appropriate action to take especially in rural communities where expert medical help may not be within immediate reach.
